# Determinants of peripheral neuropathy among diabetic patients under follow-up in chronic care clinics of public hospitals at Gamo and Gofa zones, southern Ethiopia

**DOI:** 10.1371/journal.pone.0246722

**Published:** 2021-02-16

**Authors:** Teshale Fikadu Gebabo, Tadiwos Hailu Zewdie, Sewunet Sako Shagaro, Firehiwot Haile

**Affiliations:** 1 School of Public Health, College of Medicine and Health Science, Arba Minch University, Arba Minch, Ethiopia; 2 School of Medicine, College of Medicine and Health Science, Arba Minch University, Arba Minch, Ethiopia; West Virginia University, UNITED STATES

## Abstract

**Background:**

Peripheral neuropathy is a leading cause of morbidity and increased mortality among diabetic patients. It is characterized by significant deficits in vibration and tactile sensation. With an annual incidence of 2%, it affects as many as 110 million people worldwide. The aim of this study was to assess factors associated with peripheral neuropathy among diabetic patients in chronic care clinic in Gamo and Gofa zone, South Ethiopia.

**Methods:**

An institution- based unmatched case control study was employed among 528 randomly selected participants using a pre-tested, interviewer-administered, and structured questionnaire. Bivariate and multivariable logistic regression analysis was conducted to identifiy determinants of peripheral neuropathy using IBM SPSS version 25.

**Result:**

The odds of being an urban dweller was 2.67 times higher among cases than controls [AOR = 2.67 (1.27, 5.63)]. The likelihood of fasting blood glucose level between 203 and 282 and 282 and above was 2.55 and 3.88 times higher among cases than controls [AOR = 2.55 (1.91, 7.16)] and [AOR = 3.88 (1.42, 10.60)] respectively. The probability of living with diabetes mellitus for 10 and more years was 3.88 times higher among cases than controls [AOR = 3.88 (1.42, 10.60)]. The odds of controlling glucose level after developing symptom was 5.33 times higher among cases than controls [AOR = 5.33 (1.28, 12.24)]. The probability of having high blood pressure was 2.36 times higher among cases than controls [AOR = 2.36 (1.26, 4.43)]. The likelihood of having a family history of complication from diabetes mellitus was 5.60 times higher among cases than controls [AOR = 5.60 (2.03, 15.43)]. The odds of exercising 3 times per week for 15 to 30 minutes and for less than 15 minutes were 2.96 and 4.92 times higher among cases than controls respectively [AOR = 2.96 (1.32, 6.61)] and AOR = 4.92, 95% CI (1.85, 13.04) respectively. The likelihood of having a waist circumference greater than or equal to 40 inch was 2.72 times higher among cases than controls [AOR = 2.72 (1.07, 6.94)].

**Conclusion:**

This study showed that residence, duration of diabetic mellitus, family history of complication from diabetic mellitus, level of fasting blood glucose, method of glycemic control, having a high blood pressure/hypertension/, frequency and duration of physical activity and waist circumference were found to be determinants of peripheral neuropathy. Thus, the concerned health authorities and health professionals should target on these factors in their efforts to prevent peripheral neuropathy among diabetics in the study area.

## Introduction

Diabetes mellitus (DM) is one of the most widespread chronic diseases in the world [1]. Globally, about 193 million (46.5%) people with diabetes are undiagnosed; of this 81.1% of all undiagnosed people live in low- and middle-income countries. In the Sub-Saharan Africa, the region with lack of resources and least government priority, an estimated 66.7% live with undiagnosed DM cases [2]. According to International Diabetes Federation (IDF), it is estimated that the number of adult patients with DM in the world will increase from 415 million to 642 million by the year 2040. In sub-Saharan Africa, there will be a disproportionate increment of 109.6% compared to the global increment of 55% by 2030 [2, 3]. About 75% of estimated DM cases globally live in low and middle income countries, out of which, 77.3% (320.5 million) people are in the working age group [2]. According to the 2000 WHO projection report, the number of diabetic patients in Ethiopia will be more than double by the year 2030 [3].

More than 321,100 deaths in Africa were attributed to diabetes with the highest proportion (79%) of the death being among the working age groups, under the age of 60. By the year 2010, the overall prevalence of DM was 6.5%. Of this, 15.7% to 29.5% diabetic patients developed complications [4, 5].

DM can affect various parts of the body with varying manifestations among different people. These complications can lead to disability, reduced quality of life and premature death. Diabetic peripheral neuropathy (DPN) is a leading cause of morbidity, mortality which is characterized by significant deficits in tactile sensitivity, and vibration sense. It also affects all peripheral nerves with an annual incidence of 2%. Out of 220 million estimated global cases of diabetes by the year 2010, DPN is likely to affect as many as 110 million people worldwide [6, 7].

DPN is the earliest and most common long-term complications of DM which is estimated to affect 30–50% of individuals with diabetes [8]. Studies revealed that the magnitude of DPN was 5.8–34% in Europe [9], 40.3% to 42.2% in Germany [10], 72.2% in Tanzania [11], 29.4% in Uganda [12], and 49.4% in the West Africa. The overall pooled prevalence was 46% in Africa [13] and 53.6% in Ethiopia [14]. DPN predisposes to diabetic foot ulceration (FU) [15] with a life-time risk of amputation to be 15% [1]. Due to low awareness and suboptimal management of the disease, the prevalence of long-term complications of diabetes will increase accordingly. Diabetic nephropathy is the commonest among the chronic micro vascular diabetes complications [16]. Among children and adolescents, chronic complications of DM can lead to early death, difficulty in coping emotionally with their disease, discrimination and limits social relationships. It also has an impact on a child’s academic performance, and the financial burden due to costs of treatment and monitoring equipment [1].

In Africa, about 35% of DM patients develop chronic complication within 3 years of diagnosis and 18% die of the complications after 20 years of diagnosis. Increase in prevalence of diabetes complications in Africa was attributed to the rise in financial health expenditure, poor medical facility, and lack of adequate diabetes service in urban and rural areas [17, 18].

As part of non-communicable disease prevention and treatment strategies, DPN and related complications are getting attention at the national and international levels. Guidelines are recommending a multifactorial approach in treating and preventing DPN. Regular screening program should be conducted at primary health care centers through a valid point of care devices and instruments. It is also believed that addressing modifiable risk factors can significantly delay or slow the development of DPN. Besides, other policy-level interventions such as task-shifting to non-physician health workforce, advocacy, and strengthening of the surveillance and management of DPN are also recommended by WHO and other professional society guidelines [19, 20].

The consequence of diabetic peripheral neuropathy increases the burden of global diabetic foot ulcer with a prevalence of 6.3% (13% - 3%) and 7.2% in Africa [15]. In Ethiopia, 32.8% of DM patients develop diabetes related complications [21] with the prevalence of diabetic neuropathy ranging from 29.5% to 52.2% [22, 23]. Despite having some prevalence studies conducted in Ethiopia, there are limited studies on the determinants of diabetic neuropathy. Therefore, this study was aimed to identify factors associated with peripheral neuropathy among patients following diabetic clinic in Gamo and Gofa zone, southern Ethiopia.

## Materials and methods

### Study design, setting and sampling

An institution based unmatched case control study was conducted from April to May 2019 in Gamo and Gofa zones. The main towns of the two zones, Arba Minch (Gamo zone) and Sawla (Gofa zone), are located 434 kms and 455 kms respectively far south of Addis Ababa, the capital city of Ethiopia. The two zones have a total population of 2,658,345 in the year 2017/18 as projected from the 2007 Ethiopian census. There are three general and three primary hospitals providing curative, preventive and rehabilitative service for the population in the two zones.

### Population of the study

Cases were diabetic patients in the follow-up clinic that were classified as having peripheral neuropathy by the Michigan neuropathy screening tool score of either > = 7 in the history version or > 2 in the physical examination version.

Controls were diabetic patients in the follow-up clinic that were not classified as having peripheral neuropathy by the Michigan neuropathy screening tool.

#### Source population

*For cases*. All diabetic patients in the follow-up clinic with peripheral neuropathy;

*For controls*. All diabetic patients in the follow-up clinic without peripheral neuropathy.

#### Study population

*For cases*. All diabetic patients with peripheral neuropathy in the follow-up clinic at the randomly selected public hospitals;

*For controls*. All diabetic patients without peripheral neuropathy in the follow-up clinic at the randomly selected public hospitals.

### Sampling

The sample size was calculated using a two-population proportion formula of equal sample size for the two groups. Assuming 95% confidence interval, 80% power, a 33.9% expected proportion of DM patients below 40 years old who did not develop peripheral neuropathy (control) and 23% expected proportion of DM patients below 40 years old who developed peripheral neuropathy (cases), and a case to control ratio of 1:1. Based on the above assumptions, the sample size calculated was 536 (268 cases and 268 controls). Among the variables considered, age was selected as a determinant variable for peripheral neuropathy since it gave maximum sample size [22].

Controls were selected by employing a systematic random sampling technique with a proportional allocation of samples while cases were selected using consecutive sampling method.

### Data collection procedures

Michigan neuropathy screening history version questionnaire was used to collect data by four nurses working in the diabetic clinics in each hospital. Michigan neuropathy screening examination was done by four physicians (2 in Arba Minch, 1 in Sawula and 1 in Chencha hospitals) to identify cases and controls [24]. The data were collected using a pre-tested, interviewer-administered, and structured questionnaire which addressed socio demographic, clinical and behavioral factors.

### Measurement

**In the history questionnaire,** responses were added to obtain a total score. Each responses of “Yes” to items 1–3, 5–6, 8–9, 11–12, 14–15 were counted as 1 point. “No” response for items 7 and 13 were counted as 1 point. Item #4, a measure of impaired circulation and item #10, a measure of general asthenia were not included in the scoring. A score of = >7 was considered as having peripheral neuropathy.

#### Physical assessment

*Foot inspection*. The feet were inspected for evidence of excessively dry skin, callous formation, fissures, frank ulceration or deformities. Deformities include flat feet, hammer toes, overlapping toes, hallux valgus, joint subluxation, prominent metatarsal heads, medial convexity (Charcot foot) and amputation.

*Vibration sensation*. Vibration sensation assessment was performed with the great toe unsupported. Vibration sensation was tested bilaterally using a 128 Hz tuning fork placed over the dorsum of the great toe on the boney prominence. Patients, whose eyes were closed, were asked to indicate when they can no longer sense the vibration from the vibrating tuning fork. Vibration was scored as 1) present if the examiner sensed the vibration on his or her finger for < 10 seconds, 2) reduced if sensed for ≥ 10 seconds or 3) absent (no vibration detected).

*Muscle stretch reflexes*. The ankle reflexes were examined using an appropriate reflex hammer. If the reflex was obtained, it was graded as present. If the reflex was absent, the patient was asked to perform the Jendrassic maneuver. Reflexes elicited with the Jendrassic maneuver alone were designated “present with reinforcement.” If the reflex was absent, even in the face of the Jendrassic maneuver, the reflex was considered absent.

*Monofilament testing*. The filament was applied perpendicularly and briefly with even pressure. When the filament bends, the force of 10 grams has been applied. The patient, whose eyes were closed, was asked to respond yes if he/she feels the filament. Eight correct responses out of 10 applications were considered normal: one to seven correct responses indicate reduced sensation and no correct answers were translated into absent sensation.

A score of ≥ 2 in physical assessment and/or Score of ≥7 in history questionnaire was considered as having peripheral neuropathy [24, 25].

#### Data processing

Training for data collectors was given intensively on the spot by including evaluation of their performance in assuring consistency of the data. Data were checked for completeness, edited, coded and entered into Epi data version 3.1 and exported to SPSS 25.0 statistical software for analysis. After cleaning the data for inconsistencies and missing values, descriptive statistics such as mean, frequency and percentage were calculated and data were presented using text and charts.

Bivariate analysis was done and all explanatory variables which have an association with the outcome variable at p-value less than 0.25 in bivariate analysis were included in the multivariable analysis model. Then, a multivariable analysis was employed using backward LR to determine independent predictors. Odds ratio with its 95% CI was used to decide whether those independent variables included in the multivariable analysis were statistically significant or not.

### Ethical consideration

The ethical approval letter was obtained from Arba Minch university ethical review board. Letter of cooperation was obtained from the respective hospitals and written informed consent was obtained from the study participants after informing the purpose of the study.

## Result

### Socio-demographic characteristics

Five hundred twenty-eight study participants (264 cases and 264 controls) were included in the study with 98.5% response rate. The mean age of the participants’ was 48 ± 12 years (50 years for cases and 46 years for controls). Majority of the cases, 163 (61.7%), and controls, 159 (60.2%) were male. One hundred seventy-eight (67.4%) cases and 183 (69.3%) controls were between 40 and 65 years of age. One hundred ninety-five (73.9%) cases and 171 (64.8%) controls were urban dwellers, while most cases, 226 (85.6%) and controls, 241 (91.3%) were married (**Table 1**).

**Table 1:** Socio demographic characteristics of diabetic patients under follow-up in clinics at Gamo and Gofa zones, southern Ethiopia.

Variable	Case No (%)	Control No (%)	P-value
**age category**			
< 40 years	43 (16.3)	64 (24.2)	
40 to 65 years	178 (67.4)	183 (69.3)	0.044
65 and above years	43 (16.3)	17 (6.4)	
**Sex of the respondent**			
Male	163 (61.7)	159 (60.2)	0.721
Female	101 (38.3)	105 (39.8)	
**Residence**			
urban	195 (73.9)	171 (64.8)	**0.024**
rural	69 (26.1)	93 (35.2)	
**Religion**			
Muslim	43 (16.3)	34 (12.9)	0.629
Orthodox	88 (33.3)	89 (33.7)	
Protestant	133 (50.4)	141 (53.4)	
**Educational status**			
Informal education	63(23.9)	54(20.5)	
Primary education	45(17.0)	41(15.5)	0.28
Secondary and above	156(59.1)	169(64.0)	
**Marital status**			
Married	226 (85.6)	241 (91.3)	
Single	20 (7.6)	18 (6.8)	0.011
Divorced/widowed	18 (6.8)	5 (1.9)	
**Occupation**			
Governmental worker	104 (39.4)	113 (42.8)	
Farmer	52 (19.7)	37 (14.0)	0.54
Merchant	68 (25.8)	82 (31.1)	
House wife	28 (10.6)	25 (9.5)	
Student	12 (4.5)	7 (2.7)	

### Medical history of the participants

Almost 1/3 of cases and controls were diagnosed and lived with DM for more than 10 years. Regarding the participants’ plasma glucose level, 1/4 of the cases and controls had fasting blood glucose of over 282 mg/dl. Majority of the cases and controls had a history of regular checkup. One hundred sixty (60.6%) cases and 193 (73.1%) controls had a normal blood pressure (**Table 2**).

**Table 2:** Medical history of DM patients under follow up in clinics at Gamo and Gofa zones, southern Ethiopia.

Variable	Case No (%)	Control No (%)	P-value
Type of DM			
Type 1	80 (30.3)	83 (31.4)	0.77
Type 2	184 (69.7)	181 (68.6)	
Type of medication used			
Insulin	146 (55.3)	152 (58.7)	0.435
Oral hypoglycemic drug	118 (44.7)	107 (41.3)	
Duration of DM			
1–3 years	53 (20.1)	91(34.5)	0.002
4–6 years	70 (26.5)	45 (17.0)	
7–9 years	56 (21.2)	57 (21.6)	
10 years and above	85 (32.2)	71 (26.9)	
Fasting blood glucose level			
less than 123	27 (10.2)	51 (19.3)	0.008
123 to 203	100 (37.9)	103 (39.0)	
203 to 282	71 (26.9)	51 (19.3)	
282 and above	66 (25.0)	59 (22.3)	
Method of glycemic control			
By regular check-up	259 (98.1)	244 (96.2)	
After sign/symptom	5 (1.9)	20 (3.8)	0.004
Family history of DM			
Yes	118 (44.70)	133 (50.4)	0.721
No	146 (55.3)	131 (49.6)	
Family history of DM complication			
Yes	22 (18.6)	10 (7.5)	0.01
No	96 (81.4)	123 (92.5)	
Having high blood pressure			
Yes	104 (39.4)	71 (26.9)	0.002
No	160 (60.6)	193 (73.1)	
Having comorbidity			
Yes	91(34.5)	101(38.3)	0.36
No	173(65.5)	163(61.7)	

### Behavior related characteristics of the study participants

Almost all cases and controls did not ever drink alcohol. Majority of cases and controls were currently involved in physical activities. Obesity was observed in 31 (11.7%) of cases and 11 (4.2%) of controls and 39(14.8%) cases and 15(5.7%) controls had waist circumference of more than 40 inches (**Table 3**).

**Table 3:** Behavior related characteristics of diabetic patients under follow-up in clinics at Gamo and Gofa zones, southern Ethiopia.

Variable	Case No (%)	Control No (%)	P-value
Ever drink alcohol			
Yes	14 (5.3)	4 (1.5)	0.024
No	250 (94.7)	260 (98.5)	
Involved in physical activity			
Yes	227 (86.0)	242 (91.7)	0.040
No	37 (14.0)	22 (8.3)	
Types of physical activity			
Fast Walking	154(67.8)	162(66.9)	0.92
Running	50(22.0)	56(23.1)	
Fast waking and running	23(10.1)	24(9.9)	
Duration of physical activities (3 times/week)			
45 and above minutes	38 (16.7)	60 (24.8)	0.031
30 to 45 minutes	58 (25.6)	73 (30.2)	
15 to 30 minutes	78 (34.4)	71 (29.3)	
Less than 15 minutes	53 (23.3)	38 (15.7)	
Waist circumference			
Less than 35 inches	144 (54.5)	164 (62.1)	0.004
35 to 40 inches	81 (30.7)	85 (32.2)	
40 inches and above	39 (14.8)	15 (5.7)	
Body mass index			
Normal weight	119 (45.1)	159 (60.2)	
Pre-obesity/overweight/	114 (43.2)	94 (35.6)	0.001
Obesity	31 (11.7)	11 (4.2)	

### Determinants of peripheral neuropathy

The odds of being urban dwellers was 2.67 times higher among cases than controls as compared to being rural dweller [AOR = 2.67, 95%CI (1.27, 5.63)]. The likelihood of fasting blood glucose level of 203 to 282 mg/dl and greater than or equal to 282 mg/dl were 2.55 and 3.88 times more among cases than controls when compared with fasting blood glucose level of less than 123 mg/dl [AOR = 2.55, 95%CI (1.91, 7.16)] and [AOR = 3.88, 95%CI (1.42, 10.60)] respectively. The probability of living with DM for greater than or equal to 10 years was 3.88 times higher among cases than controls as compared to living with DM for 1 to 3 years [AOR = 3.88, 95%CI (1.42, 10.60)]. The odds of controlling glucose level after developing symptom were 5.33 times higher among cases than controls when compared with controlling glucose level regularly [AOR = 5.33 95% CI (1.28, 12.24)]. The probability of having a high blood pressure was 2.36 times higher among cases than controls as compared to not having high blood pressure [AOR = 2.36, 95% CI (1.26, 4.43)]. The likelihood of having a family history of DM complication was 5.60 times higher among cases than controls as compared to not having a family history of DM complication [AOR = 5.60, 95% CI (2.03, 15.43)]. The odds of being involved in physical activities 3 times per week for 15 to 30 minutes and less than 15 minutes were 2.96 and 4.92 times higher among cases than controls when compared with being involved in physical activities three times per week for greater than or equal to 45 minutes [AOR = 2.96, 95% CI (1.32, 6.61)] and AOR = 4.92, 95% CI (1.85, 13.04) respectively. The likelihood of having a waist circumference of greater than or equal to 40 inches was 2.72 times higher among cases than controls as compared to having waist circumference less than 35 inches [AOR = 2.72, 95% CI (1.07, 6.94)] (**Table 4**).

**Table 4:** Independent predictors of peripheral neuropathy among diabetic patients under follow-up in clinics at public hospitals of Gamo and Gofa zones, southern Ethiopia.

Variable	Case No (%)	Control No (%)	COR (95% CI)	AOR (95% CI)
Residence				
urban	195 (73.9)	171 (64.8)	**1.54 (1.06, 2.23)**	**2.67 (1.27, 5.63)**
rural	69 (26.1)	93 (35.2)	1	**1**
Fasting blood glucose level				
less than 123	27 (10.2)	51 (19.3)	1	1
123 to 203	100 (37.9)	103 (39.0)	1.83 (1.07, 3.15)	1.50 (0.48, 4.73)
203 to 282	71 (26.9)	51 (19.3)	**2.63 (1.46, 4.74)**	**2.55 (1.91, 7.16)**
282 and above	66 (25.0)	59 (22.3)	**2.11 (1.18, 3.79)**	**3.88 (1.42, 10.60)**
Duration of DM				
1–3 years	53 (20.1)	91 (34.5)	1	1
4–6 years	70 (26.5)	45 (17.0)	**2.67 (1.61, 4.43)**	1.51 (0.48, 4.73)
7–9 years	56 (21.2)	57 (21.6)	**1.69 (1.02, 2.78)**	2.55 (0.91, 7.17)
10 and above years	85 (32.2)	71 (26.9)	**2.06 (1.29, 3.26)**	**3.88 (1.42, 10.60)**
Method of glycemic control				
By Regular checkup	259 (98.1)	244 (96.2)	1	1
After sign/symptom	5 (1.9)	20 (3.8)	**4.25 (1.57, 11.49)**	**5.33 (1.28, 12.24)**
Family history of DM complication				
Yes	22 (18.6)	10 (7.5)	**2.82 (1.28, 6.23)**	**5.60 (2.03, 15.43)**
No	96 (81.4)	123 (92.5)	1	1
Having high blood pressure				
Yes	104 (39.4)	71 (26.9)	**1.77 (1.22, 2.55)**	**2.36 (1.26, 4.43)**
No	160 (60.6)	193 (73.1)	1	1
Duration of physical activity (3 times/week)				
45 and above minutes	38 (16.7)	60 (24.8)	1	1
30 to 45 minutes	58 (25.6)	73 (30.2)	1.25 (0.73, 2.13)	1.67 (0.77, 3.61)
15 to 30 minutes	78 (34.4)	71 (29.3)	**1.73 (1.03, 2.91)**	**2.96 (1.32, 6.61)**
less 15 minutes	53 (23.3)	38 (15.7)	**2.20 (1.23, 3.94)**	**4.92 (1.85, 13.04)**
Waist circumference				
Less than 35 inches	144 (54.5)	164 (62.1)	1	1
35 to 40 inches	81 (30.7)	85 (32.2)	1.08 (0.74, 1.58)	1.02 (0.54, 1.92)
40 inches and above	39 (14.8)	15 (5.7)	**2.96 (1.57, 5.59)**	**2.72 (1.07, 6.94)**

## Discussion

Peripheral neuropathy is a common problem among diabetic patient that leads to unnecessary morbidity and substantial health care costs. Till to date, different intervention strategies were used to reduce this problem but its magnitude is high. Thus, this study attempted to assess the determinants of peripheral neuropathy among diabetic patients in the follow-up clinic. The findings might help in designing preventions and intervention strategies to reduce the incidence of the condition at local and national levels.

In this study, the odds of being an urban dweller were higher among cases than controls as compared to being rural dweller. It is a known fact that most individuals of urban dwellers practice sedentary life style and this in turn, may lead them to diabetic peripheral neuropathy. This finding was consistent with a study conducted in sub-Saharan Africa countries [26] but inconsistent with a study conducted in Sri Lanka [27]. This discrepancy might be due to the study design or inaccessibility of health facilities to diagnose and control diabetic complications in a rural area.

The probability of having high fasting blood glucose level was higher among cases than controls as compared to having low fasting blood glucose level in the current study. This might be because of the exposure to high blood glucose level increases the risk of developing macro vascular and micro vascular complications. Especially, a good glycemic control for the first year of diagnosis highly reduces the risk of complications [28]. This finding is consistent with cross sectional studies conducted in Iran and Egypt [1, 29] but it is inconsistent with studies conducted in Pakistan and India [30, 31]. The difference might be due to the study design, and difference in health seeking behavior.

The odds of living with DM for greater than or equal to 10 years were higher among cases than controls as compared to living with DM for 1 to 3 years. Living with the diseases for long period might increase the risk of exposure to unhealthy lifestyle and unhealthy food. For DM patient these conditions increase the risk of developing complications. This finding is similar with different studies conducted in developing and developed nations [1, 22, 27, 29, 30, 32–36]; a systematic review and meta-analysis also showed a similar finding [37].

The likelihood of controlling the level of blood glucose after developing sign or symptoms was higher among cases than controls when compared with controlling regularly. This might be attributed to the fact that a regular follow up is an important measure for early detection of change in blood glucose level. Uncontrolled blood glucose level leads to other acute and chronic complications including peripheral neuropathy [27]. This finding is in line with a study conducted in Jordan [34].

The probability of having a high blood pressure was higher among cases than controls as compared to not having a high blood pressure. Both high blood pressure and hyperglycemia are the end results of the metabolic syndrome and share common pathway that interact and influence each other [38]. A meta-analysis showed that a 20 mmHg increments in systolic blood pressure increases the risk of DM by 58% and 10 mmHg raise in diastolic blood pressure increases the risk of diabetes by 52% [39] consequently increasing the risk of developing diabetic complications. This is in line with studies conducted in Egypt, United Arab Emirates, China and Jordan [1, 33–35, 39]. Yet, no association was observed in a study conducted in Brazil [35]. This disparity might be attributed to differences in the data source used, study design and measurements.

The likelihood of having a family history of DM complications was higher among cases than controls as compared to not having a family history of DM complication. This is in line with studies conducted in Jordan and Egypt [1, 34]. This might be because of the link between DM and heredity. The odds of exercising physical activities 3 times per week for 15 to 30 minutes was higher among cases than controls when compared with exercising physical activities 3 times per week for greater than or equal to 45 minutes. Regular physical activities contribute to weight loss, improves well-being and considerable health benefits by improving cardiovascular fitness, muscle strength and insulin sensitivity [40]. These physiological changes have an effect on the development of DM and its macro and micro complications. Some studies revealed that less physical activity is associated with diabetic peripheral neuropathy [22, 34, 41]. Our study shows that the length of physical activities is important for the prevention of diabetic peripheral neuropathy which is in line with a study conducted in Jordan [34].

The likelihood of having waist circumference greater than or equal to 40 inches was higher among cases than controls as compared to having waist circumference less than 35 inches. This might be due to the fact that central obesity, assessed in this study by a waist circumference, is one of the modifiable risk factors of metabolic syndrome like diabetes. This finding is consistent with a study conducted in Korea [42].

## Conclusion and recommendation

The occurrence of peripheral neuropathy limits quality of life. Therefore, public and clinical experts should consider the prevention of peripheral neuropathy among diabetic patients through easy to apply screening tools and early identification of modifiable risk factors. In this study, non-modifiable factors like residence, duration of DM and family history of DM complication and modifiable factors like the level of fasting blood glucose, method of glycemic control, high blood pressure /hypertension, length of physical activities 3 times per week and waist circumference were independently associated with peripheral neuropathy. Thus, concerned health authorities and health professionals should strengthen the existing regular follow up, control of blood glucose level, and blood pressure measurement. Moreover, they should encourage patients to do physical exercise regularly at least for 45 minutes and decrease central obesity.

### Limitation

The study depends on past events so that findings would be subjected to recall bias and also it was difficult to ascertain whether the neuropathy was secondary to diabetes or not.

## Supporting information

S1 Questionnaire (DOCX)

## Abbreviation

AOR: Adjusted odds ratio
CI: confidence interval
CSA: central statistics agency
DM: Diabetes mellitus
DPN: diabetic peripheral neuropathy
IDF: Diabetes Federation
OR: odds ration
SPSS: statistical package for social science
WHO: world health organization
