# Supplementary material for: Determinants of peripheral neuropathy among diabetic patients under follow-up in chronic care clinics of public hospitals at Gamo and Gofa zones, southern Ethiopia
Source: PLoS One. 2021 Feb 16;16(2):e0246722. doi: 10.1371/journal.pone.0246722 (PMC7886204; doi:10.1371/journal.pone.0246722)
Supplement: S1 Questionnaire — (DOCX) [file pone.0246722.s001.docx]

**Questioner**

**Patient Version**

**MICHIGAN NEUROPATHY SCREENING INSTRUMENT**

1. **History** (To be completed by the person with diabetes)

Answer the following questions about the feeling in your legs and feet. Check yes or no based on how you usually feel. Thank you.

| 1. | Are you legs and/or feet numb? | Yes | No |
| --- | --- | --- | --- |
| 2. | Do you ever have any burning pain in your legs and/or feet? | Yes | No |
| 3. | Are your feet too sensitive to touch? | Yes | No |
| 4. | Do you get muscle cramps in your legs and/or feet? | Yes | No |
| 5. | Do you ever have any prickling feelings in your legs or feet? | Yes | No |
| 6. | Does it hurt when the bed covers touch your skin? | Yes | No |
| 7. | When you get into the tub or shower, are you able to tell the |  |  |
|  | hot water from the cold water? | Yes | No |
| 8. | Have you ever had an open sore on your foot? | Yes | No |
| 9. | Has your doctor ever told you that you have diabetic neuropathy? | Yes | No |
| 10. | Do you feel weak all over most of the time? | Yes | No |
| 11. | Are your symptoms worse at night? | Yes | No |
| 12. | Do your legs hurt when you walk? | Yes | No |
| 13. | Are you able to sense your feet when you walk? | Yes | No |
| 14. | Is the skin on your feet so dry that it cracks open? | Yes | No |
| 15. | Have you ever had an amputation? | Yes | No |

Total:

**MICHIGAN NEUROPATHY SCREENING INSTRUMENT**

1. **Physical Assessment** (To be completed by health professional)
2. Appearance of Feet

|  |  |  | **Right** |  |  |  |  |  |  | **Left** |  |  |  |
| --- | --- | --- | --- | --- | --- | --- | --- | --- | --- | --- | --- | --- | --- |
|  | a. Normal | | 0 Yes | |  | 1 No |  | Normal | | 0 Yes | 1 No | | |
|  | b. If no, check all that apply: | | | | | |  | If no, check all that apply: | | |  |  |  |
|  | Deformities | |  |  |  |  |  | Deformities | |  |  |  |  |
|  | Dry skin, callus | | |  |  |  |  | Dry skin, callus | | |  |  |  |
|  | Infection | |  |  |  |  |  | Infection | |  |  |  |  |
|  | Fissure | |  |  |  |  |  | Fissure | |  |  |  |  |
|  | Other | |  |  |  |  |  | Other | |  |  |  |  |
|  | specify: |  |  |  |  |  |  | specify: |  |  |  |  |  |
|  |  |  |  |  | **Right** | |  |  |  | **Left** |  |  |  |
| 2. | Ulceration | | Absent | |  | Present | |  | Absent | | Present | | |
|  |  |  |  | 0 |  |  | 1 |  |  | 0 |  | 1 |  |
|  |  |  |  |  | Present/ | |  |  |  | Present/ |  |  |  |
| 3. | Ankle Reflexes | | Present | Reinforcement | | | Absent | Present | | Reinforcement | | Absent | |
|  |  |  | 0 |  | 0.5 | | 1 | 0 | | 0.5 |  | 1 | |
| 4. | Vibration | | Present |  | Decreased | | Absent | Present | | Decreased | | Absent | |
|  |  |  | 0 |  | 0.5 | | 1 | 0 | | 0.5 |  | 1 | |
|  | perception at | |  |  |  |  |  |  |  |  |  |  |  |
|  | great toe | |  |  |  |  |  |  |  |  |  |  |  |
| 5. | Monofilament | | Normal |  | Reduced | | Absent | Normal | | Reduced |  | Absent | |
|  |  |  | 0 |  | 0.5 | | 1 | 0 | | 0.5 |  | 1 | |

Signature:_____________________________ Total Score _________________/10 Points

***Other acute complication mention _________________________________________

***Other chronic complication mention _________________________________________

**Part I socio demographic character**

101 Weight ________kg

102 Height ________meter

103. Waist circumference __________ inch

| ID | Age | Sex | Residence | Religion | Educational status | Marital status | Occupational status | Family size | Monthly Income |
| --- | --- | --- | --- | --- | --- | --- | --- | --- | --- |
| 103-109 |  |  |  |  |  |  |  |  |  |

**Sex:**

1. Male
2. Female

**Marital status:**

1. Married
2. Single
3. Divorced
4. Widowed

**Educational status:**

1. Illiterate
2. Read & write only
3. 1^st^ cycle (1-4)
4. 2^nd^ cycle (5-8)
5. Secondary (9-10)
6. Preparatory (11-12)
7. 12+

**Religion:**

1. Muslim
2. Orthodox
3. Protestant
4. Catholic
5. Other specify

**Occupational status:**

1. Governmental worker
2. Farmer
3. Merchant
4. House wife
5. Student
6. other specify__

**Residence:**

1. Urban
2. Rural

| PART II CLINICAL RELATED CHARACTERSTICS | | | | |
| --- | --- | --- | --- | --- |
| No | Questions | Possible choices/Answers | | skip Remark |
| 201 | Diabetic type | 1. Type 1 2. Type 2 | |  |
| 202 | Did you under DM medication | 1. Yes 2. No | |  |
| 203 | If yes type of medication you taken | 1. Insulin 2. Oral hypoglycemic drug | |  |
| 204 | For how long did you with DM | _______ years | |  |
| 205 | Fasting Blood glucose level | ________ | |  |
| 206 | Method of glycemic control | 1. Regular checkup 2. After sign/symptom 3. Not control mechanism | |  |
| 207 | Is their family history of DM in you sibling or parent | 1. Yes 2. No | |  |
| 208 | Did you discussed about the condition with in the family | 1. Yes 2. No | |  |
| 208 | If yes Is their family history of DM complication | 1. Yes 2. No | |  |
| 209 | If yes type of complication | 1. Acute 2. Chronic | |  |
| 210 | If chronic complication which one | 1. Neuropathy 2. Retinopathy 3. Nephropathy 4. Diabetic foot ulcer 5. Other | |  |
| 211 | Blood pressure | 1. _______ systolic 2. _______ diastolic | |  |
| 212 | Do you have diagnosed hypertension | 1. Yes 2. No | |  |
| 213 | If yes for how long | _______year | |  |
| 214 | If yes who did you control | 1. Medication 2. Physical activities 3. Diet modification 4. Other specify | |  |
| 215 | Did you have known chronic health problem | 1. Yes 2. No | |  |
| 216 | If yes type of the problem | 1. Cardio vascular 2. Other | |  |
| PART III BEHAVIORAL RELATED CHARACTERSTICS | | | | |
| No | Questions | | Possible choices/Answers | skip Remark |
| 301 | Do you smoke ever? | | 1. Yes 2. No |  |
| 302 | for how long did you smoke | | __________months/years |  |
| 303 | Do you smoke currently | | 1. Yes 2. No |  |
| 304 | If yes how often | | 1. Daily 2. Occasionally |  |
| 305 | How many cigarettes do/did you smoke? | | ____/day ______/week |  |
| 306 | Do you ever drink alcohol? | | 1. Yes 2. No |  |
| 307 | for how long did you drink alcohol | | ________ months/years |  |
| 308 | Do you drink alcohol currently | | 1. Yes 2. No |  |
| 309 | If yes how often | | 1. ______times/week 2. Occasionally |  |
| 311 | Did you involve in Physical activity | | 1. Yes 2. No |  |
| 312 | List the types of Physical activity you regularly done | | _____________ |  |
| 313 | How often do you involve in Physical activity | | ____times/week |  |
| 314 | For how long did you do Physical activity | | _______ minute |  |
